# General Civil Hospital, Colombo, Ceylon

**Published:** 1893-02-11

**Authors:** 


### GENERAL CIVIL HOSPITAL, COLOMBO, CEYLON.

This hospital, built and maintained by Government, is situated in one of the loveliest parts of Colombo; it is adjacent to the Cinnamon Gardens about two miles from the Fort. The buildings stand in their own grounds, or " compound," twelve acres in extent. The drive of about a hundred yards, from the entrance to the administrative block, is bordered on either side by hedges of shoe-flowers, large bushes covered with beautiful blossoms of various colours. The rest of the grounds form fine lawns, with a few trees here and there.

The buildings consist of about ten bungalows, all on ground floor, and mainly connected by covered corridors.

The nursing is done by Roman Catholic Sisters, about eight in number, and these ladies administer medicines and apply sach bandages as are required, but they are not trained nurses. The laborious part of the work is done by native servants of both sexes; and all the night watching is left to special night orderlies.

The Sisters are lodged in a beautiful bungalow, recently enlarged, which has a verandah front and back of it, and they have also a pretty flower garden.

A Burgher gentleman is the steward, and is responsible for the food, clothing, and bedding arrangements.

The operating theatre is very fine, and is considered one of the best in the East. Various important enlargements of the buildings are contemplated, we are told.

The Planters' ward deserves a special description. It lies to the right of the drive, on entering the gate, and it was partly put up by subscriptions from planters about seven years ago; the rest of the building was paid for by a public collection in honour of Dr. Antonis, one of the ablest surgeons that Ceylon has ever produced. The ward is divided up into comfortable single rooms fitted with electric bells, iron bedsteads with chain mattresses, mosquito curtains, &c. Each room has its special w.c., and there are several separate bath-rooms. This so-called ward is quite apart from the others, and has its own kitchens where European food is prepared. There is accommodation for about nine patients, and these must be planters or any other persons who can afford to pay the fee of three rupees per diem. The Seamen's ward is part of the original foundation, and also has its own kitchen, which supplies European diets. There is a dining-room and library for the patients. There are chain mattrasses mosquito curtains, &c. The bath-rooms and w.c.'s form a separate building at a few yards distance from the ward. The charge for each patient is Rs. 1·50 per diem. All the other wards are for natives, and they are quite free.

The accident block is divided into three portions and contains accommodation for 50 patients; the bedsteads are wooden, with stuffed mattresses, and there are no mosquito curtains. Bath-rooms and w.c.'s attached by short corridor. The general kitchen supplies the native diets, which consist of rice and curry, save when anything special is ordered.

The syphilis and the general surgical wards are divided from one another by a wall, and contain accommodation for about 24 patients in each part. The wards classed under the general title of Diarrhœa wards, are divided off into medical and surgical portions, each containing about eight beds. There are separate wards for offensive cases such as gangrene, foul ulcers, cancer, &c.

The Female Medical is the finest block in the whole building, it was erected about a year ago at Government expense. The Gynœcological ward is adjacent. The block contains accommodation for about 40 patients.

The nominal " European medical ward," with room for 20 patients, is at present occupied entirely by natives.

The surgical wards, &c., are similar in arrangement to those of the male patients.

The provision thus made for all kinds of diseases is sufficient evidence that the beautiful island of Ceylon is no freer from the " ills that flesh is heir to " than our own smoke-crowned cities in England.

